# Exacerbation of suicidal risks among women during the COVID-19 crisis: Insights into epidemiological trends and intervention strategies

**DOI:** 10.1192/j.eurpsy.2025.2356

**Published:** 2025-08-26

**Authors:** S. Hmimou, S. Elkafssaoui, S. Boukhorb, O. Erefai, F. Hadrya, S. Irnat, A. Soulaymani, A. Mokhtari, H. Hami

**Affiliations:** 1 Laboratory of Biology and Health, Faculty of Science, Ibn Tofail University, Kenitra; 2 Royal School of Military Health Service; 3 Higher Institute of Nursing Professions and Health Techniques, Rabat; 4 University Hassan First of Settat, Higher Institute of Health Sciences, Health Sciences and Technologies Laboratory, Settat, Morocco

## Abstract

**Introduction:**

The COVID-19 pandemic has significantly impacted mental health globally, disproportionately affecting women. The widespread repercussions highlight the necessity to delve into the factors exacerbating these impacts and tailor effective mitigation strategies to the unique challenges faced by women.

**Objectives:**

This study aims to identify and delineate specific risk factors that have escalated suicidal behaviors among women during the COVID-19 pandemic and to suggest targeted prevention strategies that address these identified factors.

**Methods:**

Employing a narrative review approach and adhering to the PRISMA guidelines, this study systematically examined literature from **PubMed** and **Scopus** on the impact of the COVID-19 pandemic on women’s suicide rates. This review focused on studies published between January 2020 and December 2024 that explored the pandemic’s effects on women’s mental health.

**Results:**

The findings indicate a profound deterioration in mental health among women during the pandemic, characterized by a spike in depression, anxiety, post-traumatic stress disorder, and suicidal behaviors. The impact was notably severe among women facing unstable living conditions, single mothers, and those experiencing domestic violence. Social isolation emerged as a critical factor exacerbating these conditions, particularly pronounced among young women and those from socioeconomically disadvantaged backgrounds. The literature also underscores a significant increase in suicide attempts, with these groups most profoundly impacted.

**Conclusions:**

This review confirms that the pandemic has exacerbated various risk factors associated with suicidal behaviors in women, particularly due to increased domestic violence, economic instability, and increased caregiving burdens, underscoring the critical need for tailored prevention strategies that specifically address women’s unique challenges. These should include measures to protect women from domestic violence, enhance access to mental health services, and increase economic support to buffer the adverse effects of health crises on women’s mental health.

**Disclosure of Interest:**

None Declared

